# Supplementary material for: Clinical outcomes after small-incision lenticule extraction versus femtosecond laser-assisted LASIK for high myopia: A meta-analysis
Source: PLoS One. 2021 Feb 8;16(2):e0242059. doi: 10.1371/journal.pone.0242059 (PMC7870077; doi:10.1371/journal.pone.0242059)
Supplement: S1 Table — (DOCX) [file pone.0242059.s001.docx]

**S1 Table.** Surgical procedures of the 10 included studies.

| **Study or subgroup** |  | **SMILE group** | **FS-LASIK group** | |
| --- | --- | --- | --- | --- |
| Bingjie Wang [2]  2016 | Laser system | VisuMax femtosecond laser system  (Carl Zeiss Meditec) | VisuMax system for flap creation followed by Mel 80 excimer laser  (Carl Zeiss Meditec) | |
|  | Cap thickness | 100–120 μm | Flap thickness | 95 μm |
|  | Cap diameter  /Lenticule diameter | mm  / 6.1 to 6.6 mm | Falp diameter | Not mentioned |
| Bingjie Wang [3]  2015 | Laser system | VisuMax femtosecond laser system  (Carl Zeiss Meditec) | VisuMax system for flap creation followed by Mel 80 excimer laser  (Carl Zeiss Meditec) | |
|  | Cap thickness | 100–120 μm | Flap thickness | 95 μm |
|  | Cap diameter  /Lenticule diameter | mm  / 6.1 to 6.6 mm | Optical zone size | 5.75–6.50 mm |
| Likun Xia [11]  2018 | Laser system | Visumax femtosecond laser system  (Carl Zeiss, Meditec AG, Germany) | VisuMax femtosecond laser  (Carl Zeiss Meditec AG, Germany) | |
|  | Cap thickness | 110 μm | Flap thickness | 110 µm |
|  | Cap diameter  /Lenticule diameter | 7.6 mm  /6.5 mm | Falp diameter | 7.9 mm |
| Tian Han [14]  2018 | Laser system | VisuMax femtosecond laser system  (Carl Zeiss Meditec) | VisuMax system for flap creation followed by Mel 80 excimer laser  (Carl Zeiss Meditec) | |
|  | Cap thickness | 120 µm | Flap thickness | 100 µm |
|  | Cap diameter  /Lenticule diameter | 7.5 mm  /6.25 and 6.70 mm | Flap diameter | 8.5 mm |
| Congrong Jing [15]  2018 | Laser system | VisuMax femtosecond laser system  (Carl Zeiss Meditec) | VisuMax system for flap creation followed by Mel 80 excimer laser  (Carl Zeiss Meditec) | |
|  | Cap thickness | 120μm | Flap thickness | 90~110μm |
|  | Lenticule diameter | 6. 5 mm | Flap diameter | 7. 9~8. 5 mm |
| Guofu Chen [16]  2017 | Laser system | VisuMax femtosecond laser system  (Carl Zeiss Meditec) | VisuMax system for flap creation followed by Mel 80 excimer laser  (Carl Zeiss Meditec) | |
|  | Cap thickness | 110μm | Flap thickness | 110μm |
|  | Lenticule diameter | 6.2 ~ 6. 5 mm | Optical zone size | approximately 6. 2 mm |
| Xiaojing Li [17]  2015 | Laser system | VisuMax femtosecond laser system  (Carl Zeiss Meditec) | VisuMax system for flap creation  (Carl Zeiss Meditec) | |
|  | Cap thickness | 110 μm | Flap thickness | 110 μm |
|  | Cap diameter  /Lenticule diameter | 7.0 mm  /6.0 mm | Flap diameter | 8.0 mm |
| Yueming Zhou[18]  2016 | Laser system | VisuMax femtosecond laser system  (Carl Zeiss Meditec) | VisuMax system for flap creation  (Carl Zeiss Meditec) | |
|  | Cap thickness | 110μm | Flap thickness | 110μm |
|  | Cap diameter | Not mentioned | Flap diameter | Not mentioned |
| Xueyi Zhou[19]  2019 | Laser system | VisuMax femtosecond laser system  (Carl Zeiss Meditec) | VisuMax system for flap creation followed by Mel 80 excimer laser  (Carl Zeiss Meditec) | |
|  | Cap thickness | 100–120 μm | Flap thickness | 100 μm |
|  | Cap diameter  /Lenticule diameter | 7.5 mm  /6 mm | Flap diameter | 8 mm |
| Iben Bach Pedersen[20]  2014 | Laser system | VisuMax femtosecond laser system  (Carl Zeiss Meditec) | VisuMax system for flap creation followed by Mel 80 excimer laser  (Carl Zeiss Meditec) | |
|  | Cap thickness | 120 μm | Flap thickness | 110 μm |
|  | Cap diameter  /Lenticule diameter | 8.0-mm  /6.2-mm to 6.5-mm | Flap diameter | 7.9 to 9.0 mm |
| Yishan Qian [38]  2020 | Laser system | Visumax femtosecond laser system  (Carl Zeiss, Meditec AG, Germany) | VisuMax system for flap creation followed by Mel 80 excimer laser  (Carl Zeiss Meditec) | |
|  | Cap thickness | 110 μm | Flap thickness | 110 μm |
|  | Cap diameter  /Lenticule diameter | 7.0–7.5 mm  /5.8-6.5 mm | Flap diameter  /optical zone | 7.5 mm  /5.75-6.25 mm |
| Tian Han [39]  2020 | Laser system | Visumax femtosecond laser system  (Carl Zeiss, Meditec AG, Germany) | VisuMax system for flap creation followed by Mel 80 excimer laser  (Carl Zeiss Meditec) | |
|  | Cap thickness | 120 μm | Flap thickness | 110 to 120 μm |
|  | Cap diameter  /Lenticule diameter | 7.5 mm  /6.25-6.70 mm | Flap diameter | 8.5 mm |
