# Supplementary material for: Clinical outcomes after small-incision lenticule extraction versus femtosecond laser-assisted LASIK for high myopia: A meta-analysis
Source: PLoS One. 2021 Feb 8;16(2):e0242059. doi: 10.1371/journal.pone.0242059 (PMC7870077; doi:10.1371/journal.pone.0242059)
Supplement: S2 Table — (DOCX) [file pone.0242059.s002.docx]

**S2 Table.** Summary of Findings.

| **Outcomes** | **Follow up** | **Studies** | **Eyes (n)** | | **Effect measure** | **Test for heterogenicity** | **Test for overall effect:**  **Z test** |
| --- | --- | --- | --- | --- | --- | --- | --- |
|  |  |  | SMILE | FS-LASIK | WMD (95% CI) | I² | P value |
| UDVA  (Subgroups) | 1 mo | 3 | 249 | 215 | -0.01[-0.02,0.00] | 0% | 0.07 |
|  | 3 mo | 3 | 249 | 215 | -0.00[-0.01,0.01] | 0% | 0.83 |
|  | Long term | 6 | 462 | 367 | -0.00[-0.01,0.00] | 32% | 0.33 |
|  | Total | 6 | 676 | 575 | -0.00[-0.01,0.00] | 0% | 0.13 |
| The logMAR values of CDVA | Long term | 5 | 303 | 231 | -0.04[-0.05, 0.02] | 0% | **＜0.00001 *** |
| Postoperative refractive SE | Long term | 6 | 453 | 354 | -0.03[-0.09,0.03] | 13% | 0.30 |
| tHOA | Long term | 4 | 259 | 223 | -0.09[-0.10, 0.07] | 7% | **＜0.00001 *** |
| Spherical aberration | Long term | 3 | 193 | 157 | -0.15[-0.19, 0.11] | 29% | **＜0.00001 *** |
| Coma | Long term | 3 | 181 | 158 | -0.05[-0.06, 0.03] | 30% | **＜0.00001 *** |
