# Supplementary material for: Clinical outcomes after small-incision lenticule extraction versus femtosecond laser-assisted LASIK for high myopia: A meta-analysis
Source: PLoS One. 2021 Feb 8;16(2):e0242059. doi: 10.1371/journal.pone.0242059 (PMC7870077; doi:10.1371/journal.pone.0242059)
Supplement: Attachment — Submitted filename: Response_to_Reviewers.docx [file pone.0242059.s004.docx]

Response to Reviewers

Reviewer #1: This manuscript summarizes clinical outcomes after small incision lenticule extraction versus femtosecond laser assisted LASIK for high myopia using meta-analysis. I have below questions and comments.

Table 1. It’s not clearly described what the numbers are under SE(D) are. Which number is SE? What’s SE or D?
Answer: SE means postoperative mean refractive spherical equivalent, and D means diopter. All values in the “SE(D)” column represent the postoperative mean refractive spherical equivalent in diopters. We have revised “SE(D)” to “Preop mean SE (D)” and added comments below Table 1.

The funnel plot (Supplementary Figure 1) with statistical test results are not robust because tests for funnel plot asymmetry should not be used when the number of studies is less than 10.
Answer: We have removed Supplementary Figure 1.

Table 3, What CH?(P<0.001)? What are those numbers are under SMILE or FS-LASIK?
Answer: To make this manuscript clearer, we have left out biomechanical parameters including posterior corneal elevation (PCE) changes as well as CH and CRF values.

In Figures 3 and 4, please add note/legend to clearly indicate which outcome is summarized under figure A, B, C or D.
Answer: We have added notes under Figures 3 and 4.

Line 171, “All of these 10 included studies were retrospective non-random control trials”. Some of these trials are prospective trials. Please double check.
Answer: We have checked and modified it (lines 242-243).

What are the potential reasons that such trials only performed or reported from Asia?
Answer: We have added the following explanation (lines 414-416): “… the processed screening results showed that most of the included studies were performed in China, which may have caused publication bias.”

The font size is too small to review conveniently. Please increase the font size in revisions.
Answer: We have standardized the font size.

Authors please ask an English editor to edit the writing in English.

Answer: We have invited a scientific editor at American Journal Experts (AJE) to edit the English in our manuscript.

Reviewer #2: General comments:
#1 The authors have tried to perform a meta analysis on clinical outcomes of SMILE versus FS-LASIK for high myopia. Such initiatives are welcome, but unfortunately very few well-performed RCTs have been performed. Maybe just one...

Answer: We have added the following explanation (lines 413-414): “… only one randomized trial was included, which increased the risk of various types of bias.”

#2 I think the authors should focus on one outcome, maybe three clinical outcomes: Safety, efficacy and predictability, and focus their manuscript on these aspects. They should leave out biomechanics changes, aberrations etc. This will make the manuscript more clear (see Specific comments).

Answer: To make this manuscript clearer, we have left out biomechanical parameters including posterior corneal elevation (PCE) changes as well as CH and CRF values.

#3 The vast majority of included studies are based on Chinese patients. This should be discussed.

Answer: We have added the following explanation: “… the processed screening results showed that most of the included studies were performed in China, which may have caused publication bias.”

#4 The manuscript needs to be revised by a native English person.
Answer: We have invited a scientific editor at American Journal Experts (AJE) to edit the English in our manuscript.

Specific comments:
#5 Line 68: Please correct that publications up to November 2019 were included.√

PS: We performed the literature searches from March 2018 to July 2020; we did not mean to imply that the latest publication was from July 2020.

#6 Line 223 : Drop "Biomechanical effects"√
#7 Line 249 ff: Drop discussion on "glare" as this was not primary outcome. √
#8 Line 254: Drop discussion on trauma. √
#9 Line 259: Drop discussion on "wound healing"√
#10 Line 266: Drop discussion on posterior cornea, as this was not investigated. √
#11 Line 277: Drop discussion on contest sensitivity, as this was not investigated. √
#12 Line 283: Drop discussion on biomechanics changes. √
#13 Line 287: Drop discussion on PCE, as this was investigated. √

Reviewer #3: This is an important Meta Analysis, and one that has been carried out carefully by the authors. The only question i have is how the authors have left out one study in one context (for looking at UDVA) and used it in another context (HOA's).

Answer: That study (Likun Xia et al. [11]) showed an outsized influence on the results in the “leave-one-out” analysis for UDVA (I2=69% when included, but I2=0% when excluded). However, the “leave-one-out” analyses for tHOA, spherical aberration and coma did not show that those outcomes were highly sensitive to Xia et al. In addition, the results of the statistical analysis were the same whether that study was included or excluded.
