# Supplementary material for: Clinical outcomes after small-incision lenticule extraction versus femtosecond laser-assisted LASIK for high myopia: A meta-analysis
Source: PLoS One. 2021 Feb 8;16(2):e0242059. doi: 10.1371/journal.pone.0242059 (PMC7870077; doi:10.1371/journal.pone.0242059)
Supplement: Attachment — Submitted filename: Response to Reviewers.docx [file pone.0242059.s005.docx]

Response to Reviewers

Reviewer #1: (No Response)

Reviewer #2: My comments have been addressed, but the abstract have an error which needs to be corrected: In the Results section, the authors write "Pooled results revealed no significant differences ..., the logMAR of postoperative corrected distance visual acuity (CDVA; WMD=-0.04, 95% CI, -0.05 to 0.02, I2=0%, P<0.00001), ..." Think the correct 95% CI is -0.05 to -0.02, which the P value also indicate, and this is also written in the Results section in the manuscript.
Thus, the sentence needs to be corrected.

Answer: We have corrected the Results section in abstract. (line88-93)

Reviewer #3: (No Response)

In addition, we have deleted the ethics statement which is written in any other section besides the Methods according to the editor's comments.
