# Supplementary material for: Clinical outcomes after small-incision lenticule extraction versus femtosecond laser-assisted LASIK for high myopia: A meta-analysis
Source: PLoS One. 2021 Feb 8;16(2):e0242059. doi: 10.1371/journal.pone.0242059 (PMC7870077; doi:10.1371/journal.pone.0242059)
Supplement: Attachment — Submitted filename: Response to Reviewers03.docx [file pone.0242059.s006.docx]

Response to Reviewers

1) We note that your Data Availability Statement reads, "All relevant data are within the manuscript and its Supporting Information files" while your Manuscript states that "Availability of data and materials: Available upon request from the first author; Dr. Yanyan Fu."

Could you please clarify where your minimal data set can be accessed? Please note that PLOS ONE does not permit data to only be available upon request from the authors.

We define the minimal data set as that which is used to reach the conclusions drawn in the manuscript with related metadata and methods, and any additional data required to replicate the reported study findings in their entirety.

For systematic reviews and meta-analyses, meeting the Minimal Data Set requirement typically means that authors must provide detailed information regarding their search strategy and the articles that were found. Thus, we ask that you please confirm whether the information necessary to replicate the study is in the Supporting Information files. If not, please upload a file with the information necessary to allow for replication.

Answer: We have made all data underlying the findings in this manuscript fully available, and already provided detailed information regarding the search strategy (line 152-157).

2) Please amend the title either on the online submission form or in your manuscript so that they are identical.

Answer: We have amended the title.
